# 4,10,16,22-Tetra­kis(2-chloro­acet­oxy)-6,12,18,24-tetra­meth­oxy-2,8,14,20-tetra­pentyl­resorcin[4]arene

**DOI:** 10.1107/S160053681103916X

**Published:** 2011-10-12

**Authors:** Pramod B. Pansuriya, Holger B. Friedrich, Glenn E. M. Maguire

**Affiliations:** a School of Chemistry, University of KwaZulu-Natal, Durban 4000, South Africa

## Abstract

The title compound, C_60_H_76_Cl_4_O_12_, has a macrocyclic structure and both the upper and lower rim have disordered atoms. There are no hydrogen bonds or π–π stacking inter­actions in the crystal.

## Related literature

For applications of resorcin[4]arenes, see: Asadi *et al.* (2011 ▶); Yong *et al.* (2010 ▶); Balasubramanian *et al.* (2007 ▶); Misra & Liu (2007 ▶); Dickert *et al.* (1997 ▶). For structural information, see: Wiegmann & Mattay (2011 ▶). For the synthesis of tetra­meth­oxy resorcin[4]arene, see: McIldowie *et al.* (2000 ▶).
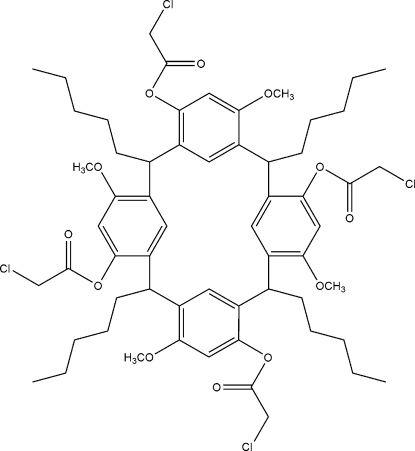


## Experimental

### 

#### Crystal data

C_60_H_76_Cl_4_O_12_
*M* *_r_* = 1131.01
Monoclinic, 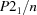

*a* = 12.4190 (5) Å
*b* = 26.1925 (11) Å
*c* = 19.1638 (8) Å
β = 107.407 (1)°
*V* = 5948.2 (4) Å^3^
*Z* = 4
Mo *K*α radiation
μ = 0.26 mm^−1^
*T* = 173 K
0.22 × 0.18 × 0.16 mm

#### Data collection

Bruker Kappa DUO APEXII diffractometer
Absorption correction: multi-scan (*SADABS*; Bruker, 2006 ▶) *T*_min_ = 0.945, *T*_max_ = 0.960
37389 measured reflections
13698 independent reflections
8473 reflections with *I* > 2σ(*I*)
*R*_int_ = 0.037

#### Refinement

*R*[*F* ^2^ > 2σ(*F* ^2^)] = 0.063
*wR*(*F* ^2^) = 0.199
*S* = 1.05
13698 reflections
694 parameters
12 restraints
H-atom parameters constrained
Δρ_max_ = 0.61 e Å^−3^
Δρ_min_ = −0.60 e Å^−3^

### 

Data collection: *APEX2* (Bruker, 2006 ▶); cell refinement: *SAINT* (Bruker, 2006 ▶); data reduction: *SAINT*; program(s) used to solve structure: *SHELXS97* (Sheldrick, 2008 ▶); program(s) used to refine structure: *SHELXL97* (Sheldrick, 2008 ▶); molecular graphics: *OLEX2* (Dolomanov *et al.*, 2009 ▶); software used to prepare material for publication: *SHELXL97*.

## Supplementary Material

Crystal structure: contains datablock(s) I, global. DOI: 10.1107/S160053681103916X/hg5096sup1.cif

Structure factors: contains datablock(s) I. DOI: 10.1107/S160053681103916X/hg5096Isup2.hkl

Additional supplementary materials:  crystallographic information; 3D view; checkCIF report

## Figures and Tables

**Fig. 1.**
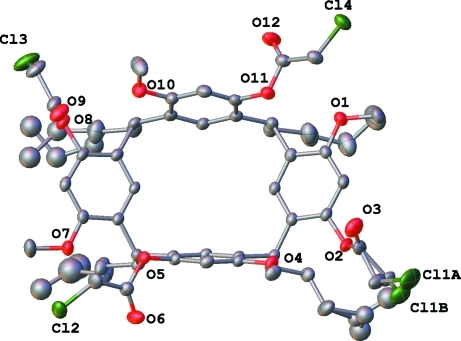
The molecular structure of the title compound with selected atomic numbering; the H atoms have been omitted for clarity. Displacement ellipsoids are drawn at 40% probability.

**Fig. 2.**
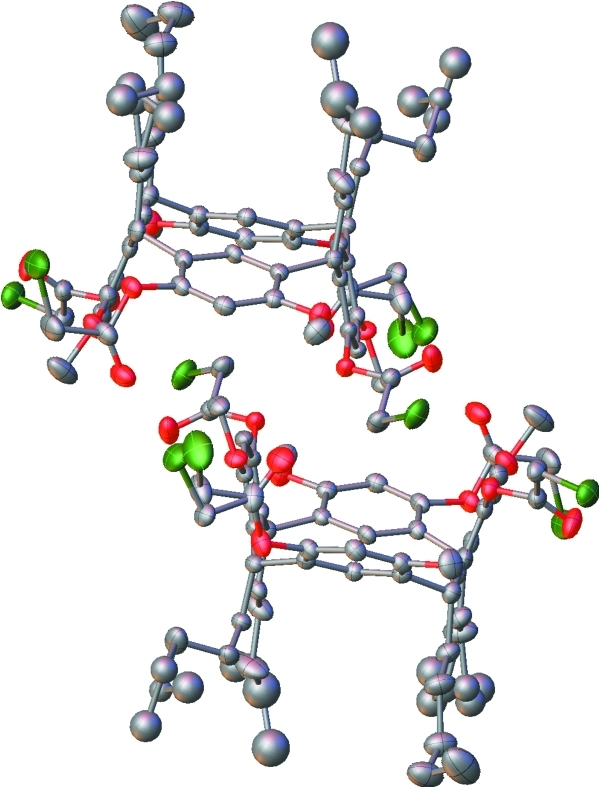
The capsule formation by two "bowl" structures. Hydrogen atoms in each unit have been omitted. Displacement ellipsoids are drawn at 40% probability.

**Fig. 3.**
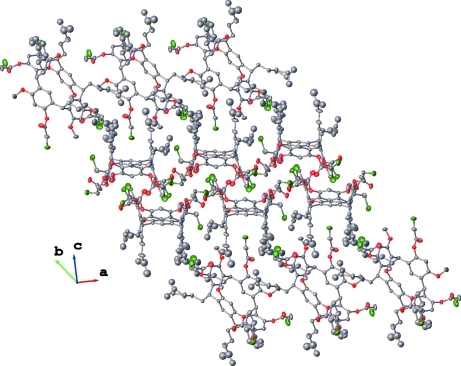
Packing of the title compound in the crystal. The interlocking of "head groups" as well as interdigitation of one of the chlorines in the "feet" is visible. All H atoms have been omitted for clarity.

## supplementary crystallographic information

### Comment

Over recent decades interest in resorcin[4]arenes has grown. They are ideal macrocyclic molecules with versatile applications such as hosts for molecular recognition (Asadi *et al.*, 2011), catalysts (Yong *et al.*, 2010), functionalization into nanoparticle scaffolds (Balasubramanian *et al.*, 2007), multidentate ligands (Misra & Liu 2007), and analytical reagents (Dickert *et al.*, 1997).

A wide range of resorcin[4]arene derivatives with different physicochemical properties have thus been synthesized. One family of these molecules which has received much attention recently is the semi flexible tetramethoxy resorcin[4]arenes (Wiegmann *et al.*, 2011). Here we report the crystal structure of novel a tetramethoxy resorcin[4]arene derivative containing chloroacetoxy moieties on the upper rim.

The title compound has a *rccc* (boat) configuration (Fig. 1). The structure posseses disorder in both the upper and lower rim regions. In the crystal the molecule forms capsule structures due to interlocking between two molecules by way of the chloroacetoxy "head groups" (Fig. 2). The feet are such that two adjacent pentyl groups are linear, while the remaining two are bent. This may be due to interdigitation of a chlorine atom in between the "feet" (Fig. 3). This results in insufficient space for the remaining pairs of alkyl groups to extend in a linear fashion. There are no hydrogen bonds or π-π stacking interactions in the crystal.

### Experimental

2,8,14,20-Tetrapentyl-4,10,16,22-tetrakis (2-chloroacetoxy)-6,12,18,24-tetramethoxyresorcin[4]arene

Tetramethoxy resorcin[4]arene (0.825 g, 1 mmol) (McIldowie *et al.* (2000)) and pyridine (0.363 g, 8 mmol) were added in tetrahydrofuran (20 ml) followed by the addition of chloroacetyl chloride (0.903 g, 8 mmol). The solution was stirred at room temperature for 24 h. Subsequently, methylene chloride (20 ml) was added and then the solvent was removed under vacuum at 60°C. Water and methylene chloride was then added to the residue. The organic layer was washed with water, dried over anhydrous magnesium sulfate and then removed under reduced presssure. The residue was then stirred in methanol, filtered and washed with methanol to give pure product (Yield: 0.90 g, 80%).

Crystals suitable for single-crystal X-ray diffraction were grown in methanol:methylene chloride(1:2)  at room temperature. M.p. = 464 K.

### Refinement

All non-hydrogen atoms, except some of the disordered moieties, were refined anisotropically. The disordered moieties are Cl1A and C31A *versus* Cl1B and C31B with site occupancy factors of 0.50 each; C35A and C36A *versus* C35B and C36B with site occupancy factors of 0.689 and 0.311 respectively; C44A *versus* C44B with site occupancy factors of 0.472 and 0.528 respectively; C50A, C51A and C52A *versus* C50B, C51B and C52B with site occupancy factors of 0.450 and 0.550 respectively. All these disordered atoms except Cl1A, Cl1B, C31A and C31B, were refined with isotropic temperature factors. C36A, C36B, C44A, C44B, C52A and C52B were refined with fixed *U*_iso_ value. All the hydrogen atoms were placed at calculated positions with bond distances to their parent atoms ranging from 0.95 Å to 1.00 Å and refined as riding on their parent atoms with *U*_iso_ (H) = 1.2 or 1.5 *U*_eq_ (C).

### Crystal data

**Table:**

C_60_H_76_Cl_4_O_12_	*F*(000) = 2400
*M**_r_* = 1131.01	*D*_x_ = 1.263 Mg m^−^^3^
Monoclinic, *P*2_1_/*n*	Melting point: 464 K
Hall symbol: -P 2yn	Mo *K*α radiation, λ = 0.71073 Å
*a* = 12.4190 (5) Å	Cell parameters from 37389 reflections
*b* = 26.1925 (11) Å	θ = 1.6–27.6°
*c* = 19.1638 (8) Å	µ = 0.26 mm^−^^1^
β = 107.407 (1)°	*T* = 173 K
*V* = 5948.2 (4) Å^3^	Block, colourless
*Z* = 4	0.22 × 0.18 × 0.16 mm

### Data collection

**Table:**

Bruker Kappa DUO APEXII diffractometer	13698 independent reflections
Radiation source: fine-focus sealed tube	8473 reflections with *I* > 2σ(*I*)
graphite	*R*_int_ = 0.037
0.5° φ scans and ω	θ_max_ = 27.6°, θ_min_ = 1.6°
Absorption correction: multi-scan (*SADABS*; Bruker, 2006)	*h* = −16→15
*T*_min_ = 0.945, *T*_max_ = 0.960	*k* = −26→34
37389 measured reflections	*l* = −24→24

### Refinement

**Table:**

Refinement on *F*^2^	Primary atom site location: structure-invariant direct methods
Least-squares matrix: full	Secondary atom site location: difference Fourier map
*R*[*F*^2^ > 2σ(*F*^2^)] = 0.063	Hydrogen site location: inferred from neighbouring sites
*wR*(*F*^2^) = 0.199	H-atom parameters constrained
*S* = 1.05	*w* = 1/[σ^2^(*F*_o_^2^) + (0.0967*P*)^2^ + 2.9849*P*] where *P* = (*F*_o_^2^ + 2*F*_c_^2^)/3
13698 reflections	(Δ/σ)_max_ = 0.001
694 parameters	Δρ_max_ = 0.61 e Å^−^^3^
12 restraints	Δρ_min_ = −0.60 e Å^−^^3^

### Special details

**Table:**

Geometry. All e.s.d.'s (except the e.s.d. in the dihedral angle between two l.s. planes) are estimated using the full covariance matrix. The cell e.s.d.'s are taken into account individually in the estimation of e.s.d.'s in distances, angles and torsion angles; correlations between e.s.d.'s in cell parameters are only used when they are defined by crystal symmetry. An approximate (isotropic) treatment of cell e.s.d.'s is used for estimating e.s.d.'s involving l.s. planes.
Refinement. Refinement of *F*^2^ against ALL reflections. The weighted *R*-factor *wR* and goodness of fit *S* are based on *F*^2^, conventional *R*-factors *R* are based on *F*, with *F* set to zero for negative *F*^2^. The threshold expression of *F*^2^ > σ(*F*^2^) is used only for calculating *R*-factors(gt) *etc*. and is not relevant to the choice of reflections for refinement. *R*-factors based on *F*^2^ are statistically about twice as large as those based on *F*, and *R*- factors based on ALL data will be even larger.

### Fractional atomic coordinates and isotropic or equivalent isotropic displacement parameters (Å^2^)

**Table:**

	*x*	*y*	*z*	*U*_iso_*/*U*_eq_	Occ. (<1)
Cl2	0.37232 (8)	1.17006 (3)	0.77801 (6)	0.0613 (3)	
Cl3	1.02976 (8)	0.94705 (5)	0.71176 (7)	0.0896 (4)	
Cl4	0.89879 (7)	0.69935 (3)	1.22023 (5)	0.0568 (2)	
O1	0.58385 (17)	0.71264 (8)	1.08755 (11)	0.0447 (5)	
O2	0.22948 (15)	0.80022 (9)	1.04907 (11)	0.0467 (5)	
O3	0.3177 (2)	0.83760 (11)	1.15675 (14)	0.0655 (7)	
O4	0.28349 (16)	0.92378 (8)	1.02051 (10)	0.0383 (4)	
O5	0.42609 (14)	1.04324 (7)	0.87884 (10)	0.0343 (4)	
O6	0.25737 (18)	1.07961 (8)	0.82508 (15)	0.0590 (6)	
O7	0.48893 (17)	1.06167 (8)	0.72073 (12)	0.0435 (5)	
O8	0.83894 (15)	0.97054 (8)	0.75480 (11)	0.0404 (5)	
O9	0.91678 (18)	1.02505 (10)	0.84722 (13)	0.0604 (7)	
O10	0.88876 (17)	0.91723 (8)	0.92280 (12)	0.0495 (5)	
O11	0.75973 (16)	0.80334 (7)	1.07790 (10)	0.0399 (5)	
O12	0.8927 (2)	0.74518 (10)	1.07721 (13)	0.0610 (7)	
C1	0.5166 (2)	0.76784 (10)	0.98675 (14)	0.0329 (6)	
C2	0.5025 (2)	0.74646 (10)	1.05024 (14)	0.0341 (6)	
C3	0.4093 (2)	0.75906 (11)	1.07244 (15)	0.0366 (6)	
H3	0.4000	0.7449	1.1159	0.044*	
C4	0.3301 (2)	0.79262 (11)	1.03034 (15)	0.0353 (6)	
C5	0.3388 (2)	0.81517 (10)	0.96691 (14)	0.0303 (5)	
C6	0.4356 (2)	0.80219 (10)	0.94770 (14)	0.0310 (5)	
H6	0.4465	0.8178	0.9056	0.037*	
C7	0.2476 (2)	0.84855 (10)	0.91776 (13)	0.0298 (5)	
H7	0.1909	0.8550	0.9444	0.036*	
C8	0.29357 (19)	0.90037 (10)	0.90445 (13)	0.0273 (5)	
C9	0.31071 (19)	0.93773 (10)	0.95898 (13)	0.0294 (5)	
C10	0.3523 (2)	0.98547 (10)	0.94921 (14)	0.0320 (6)	
H10	0.3642	1.0108	0.9863	0.038*	
C11	0.37592 (19)	0.99554 (10)	0.88454 (14)	0.0291 (5)	
C12	0.35850 (19)	0.96081 (10)	0.82739 (13)	0.0277 (5)	
C13	0.31784 (19)	0.91312 (10)	0.84009 (13)	0.0281 (5)	
H13	0.3060	0.8880	0.8029	0.034*	
C14	0.38146 (19)	0.97586 (10)	0.75646 (13)	0.0294 (5)	
H14	0.3520	1.0113	0.7443	0.035*	
C15	0.5075 (2)	0.97705 (10)	0.76416 (13)	0.0289 (5)	
C16	0.5570 (2)	1.02006 (10)	0.74239 (14)	0.0327 (6)	
C17	0.6695 (2)	1.01844 (11)	0.74282 (14)	0.0355 (6)	
H17	0.7041	1.0476	0.7292	0.043*	
C18	0.7296 (2)	0.97399 (11)	0.76317 (14)	0.0340 (6)	
C19	0.6851 (2)	0.93042 (11)	0.78424 (14)	0.0320 (6)	
C20	0.5727 (2)	0.93388 (10)	0.78544 (14)	0.0300 (5)	
H20	0.5402	0.9051	0.8017	0.036*	
C21	0.7459 (2)	0.87911 (11)	0.79701 (16)	0.0369 (6)	
H21	0.8263	0.8860	0.7996	0.044*	
C22	0.7473 (2)	0.85710 (11)	0.87009 (15)	0.0354 (6)	
C23	0.8220 (2)	0.87808 (11)	0.93367 (16)	0.0384 (6)	
C24	0.8252 (2)	0.86028 (11)	1.00241 (16)	0.0403 (6)	
H24	0.8738	0.8755	1.0454	0.048*	
C25	0.7559 (2)	0.81987 (11)	1.00677 (15)	0.0356 (6)	
C26	0.6820 (2)	0.79624 (10)	0.94673 (15)	0.0338 (6)	
C27	0.6791 (2)	0.81638 (11)	0.87832 (15)	0.0349 (6)	
H27	0.6286	0.8017	0.8357	0.042*	
C28	0.6123 (2)	0.75094 (10)	0.95753 (15)	0.0360 (6)	
H28	0.6636	0.7292	0.9964	0.043*	
C29	0.5627 (3)	0.68305 (14)	1.14442 (19)	0.0573 (9)	
H29A	0.5575	0.7057	1.1840	0.086*	
H29B	0.6245	0.6587	1.1633	0.086*	
H29C	0.4916	0.6644	1.1251	0.086*	
C30	0.2355 (3)	0.82100 (14)	1.11442 (18)	0.0504 (8)	
C32	0.1851 (2)	0.82006 (10)	0.84650 (14)	0.0341 (6)	
H32A	0.1345	0.8443	0.8124	0.041*	
H32B	0.2411	0.8076	0.8230	0.041*	
C33	0.1157 (2)	0.77491 (11)	0.85916 (17)	0.0403 (6)	
H33A	0.1646	0.7527	0.8975	0.048*	
H33B	0.0902	0.7546	0.8136	0.048*	
C37	0.2840 (2)	0.96274 (13)	1.07281 (16)	0.0439 (7)	
H37A	0.3608	0.9760	1.0933	0.066*	
H37B	0.2579	0.9484	1.1122	0.066*	
H37C	0.2337	0.9905	1.0488	0.066*	
C38	0.3572 (2)	1.08245 (11)	0.84892 (16)	0.0391 (6)	
C39	0.4290 (3)	1.12970 (11)	0.85365 (19)	0.0468 (7)	
H39A	0.5065	1.1196	0.8553	0.056*	
H39B	0.4332	1.1484	0.8993	0.056*	
C40	0.3215 (2)	0.94171 (11)	0.69113 (14)	0.0366 (6)	
H40A	0.3509	0.9065	0.7013	0.044*	
H40B	0.2399	0.9408	0.6864	0.044*	
C41	0.3361 (3)	0.95917 (16)	0.61902 (17)	0.0570 (9)	
H41A	0.3039	0.9939	0.6078	0.068*	
H41B	0.4177	0.9613	0.6242	0.068*	
C42	0.2804 (4)	0.9242 (2)	0.55562 (19)	0.0794 (13)	
H42A	0.3022	0.8887	0.5714	0.095*	
H42B	0.1977	0.9268	0.5461	0.095*	
C45	0.5285 (3)	1.10133 (12)	0.6836 (2)	0.0541 (8)	
H45A	0.5944	1.1178	0.7176	0.081*	
H45B	0.4686	1.1267	0.6654	0.081*	
H45C	0.5497	1.0868	0.6425	0.081*	
C46	0.9241 (2)	0.99649 (13)	0.80048 (17)	0.0460 (7)	
C47	1.0362 (3)	0.98543 (17)	0.7874 (2)	0.0659 (11)	
H47A	1.0861	0.9686	0.8316	0.079*	
H47B	1.0716	1.0183	0.7814	0.079*	
C48	0.6976 (3)	0.84422 (13)	0.73124 (17)	0.0483 (7)	
H48A	0.6161	0.8390	0.7242	0.058*	
H48B	0.7351	0.8105	0.7412	0.058*	
C53	0.9810 (3)	0.93181 (15)	0.9826 (2)	0.0713 (12)	
H53A	0.9532	0.9464	1.0211	0.107*	
H53B	1.0263	0.9573	0.9666	0.107*	
H53C	1.0277	0.9018	1.0017	0.107*	
C54	0.8348 (2)	0.76581 (11)	1.10790 (16)	0.0402 (6)	
C55	0.8325 (3)	0.75687 (12)	1.18448 (16)	0.0448 (7)	
H55A	0.7531	0.7561	1.1852	0.054*	
H55B	0.8709	0.7855	1.2158	0.054*	
C56	0.5662 (3)	0.71637 (12)	0.88991 (17)	0.0447 (7)	
H56A	0.5133	0.7362	0.8503	0.054*	
H56B	0.6294	0.7049	0.8723	0.054*	
C57	0.5046 (3)	0.66932 (13)	0.9078 (2)	0.0606 (9)	
H57A	0.5556	0.6519	0.9510	0.073*	
H57B	0.4378	0.6812	0.9213	0.073*	
C58	0.4657 (4)	0.63036 (15)	0.8457 (2)	0.0718 (11)	
H58A	0.5329	0.6150	0.8363	0.086*	
H58B	0.4220	0.6483	0.8006	0.086*	
C59	0.3923 (4)	0.58741 (17)	0.8626 (3)	0.0835 (13)	
H59A	0.3247	0.6030	0.8712	0.100*	
H59B	0.3661	0.5651	0.8191	0.100*	
C60	0.4499 (5)	0.5553 (2)	0.9268 (4)	0.117 (2)	
H60A	0.5198	0.5415	0.9206	0.176*	
H60B	0.4002	0.5271	0.9309	0.176*	
H60C	0.4677	0.5760	0.9713	0.176*	
Cl1A	0.0878 (2)	0.84469 (12)	1.18840 (16)	0.0715 (7)	0.50
C31A	0.1126 (9)	0.8071 (3)	1.1188 (6)	0.044 (2)	0.50
H31A	0.1085	0.7704	1.1300	0.053*	0.50
H31B	0.0553	0.8144	1.0714	0.053*	0.50
C34A	0.0121 (3)	0.79050 (14)	0.8823 (2)	0.0607 (11)	0.689 (6)
H34A	−0.0398	0.8100	0.8417	0.073*	0.689 (6)
H34B	0.0373	0.8138	0.9247	0.073*	0.689 (6)
C35A	−0.0552 (5)	0.7461 (2)	0.9031 (4)	0.0659 (16)*	0.689 (6)
H35A	−0.1054	0.7591	0.9306	0.079*	0.689 (6)
H35B	−0.0033	0.7205	0.9335	0.079*	0.689 (6)
C36A	−0.1256 (6)	0.7222 (3)	0.8289 (4)	0.090*	0.689 (6)
H36A	−0.1704	0.6936	0.8383	0.135*	0.689 (6)
H36B	−0.0745	0.7097	0.8024	0.135*	0.689 (6)
H36C	−0.1761	0.7481	0.7995	0.135*	0.689 (6)
C43A	0.3050 (6)	0.9326 (3)	0.4853 (4)	0.127 (2)*	0.472 (7)
H43A	0.3856	0.9416	0.4954	0.153*	0.472 (7)
H43B	0.2595	0.9618	0.4594	0.153*	0.472 (7)
C44A	0.2794 (12)	0.8865 (5)	0.4370 (8)	0.127 (2)*	0.472 (7)
H44A	0.2976	0.8936	0.3916	0.191*	0.472 (7)
H44B	0.1990	0.8782	0.4255	0.191*	0.472 (7)
H44C	0.3246	0.8576	0.4623	0.191*	0.472 (7)
C49A	0.7135 (4)	0.86634 (16)	0.6609 (2)	0.0706 (12)	0.450 (6)
H49A	0.6639	0.8965	0.6455	0.085*	0.450 (6)
H49B	0.7926	0.8778	0.6706	0.085*	0.450 (6)
C50A	0.6871 (10)	0.8288 (4)	0.6016 (5)	0.067 (3)*	0.450 (6)
H50A	0.6066	0.8190	0.5896	0.081*	0.450 (6)
H50B	0.7331	0.7977	0.6182	0.081*	0.450 (6)
C51A	0.7109 (10)	0.8501 (4)	0.5314 (6)	0.081 (3)*	0.450 (6)
H51A	0.6730	0.8274	0.4901	0.097*	0.450 (6)
H51B	0.7931	0.8476	0.5387	0.097*	0.450 (6)
C52A	0.6735 (10)	0.9066 (4)	0.5080 (6)	0.090*	0.450 (6)
H52A	0.6942	0.9153	0.4639	0.135*	0.450 (6)
H52B	0.7115	0.9300	0.5476	0.135*	0.450 (6)
H52C	0.5916	0.9097	0.4978	0.135*	0.450 (6)
Cl1B	0.1283 (3)	0.83185 (18)	1.21844 (16)	0.0998 (12)	0.50
C31B	0.1278 (9)	0.8330 (5)	1.1283 (6)	0.064 (3)	0.50
H31C	0.0703	0.8085	1.1005	0.076*	0.50
H31D	0.1036	0.8675	1.1083	0.076*	0.50
C34B	0.0121 (3)	0.79050 (14)	0.8823 (2)	0.0607 (11)	0.311 (6)
H34C	−0.0259	0.8203	0.8536	0.073*	0.311 (6)
H34D	0.0346	0.7995	0.9348	0.073*	0.311 (6)
C35B	−0.0698 (9)	0.7420 (4)	0.8665 (7)	0.048 (3)*	0.311 (6)
H35C	−0.1485	0.7532	0.8597	0.057*	0.311 (6)
H35D	−0.0670	0.7250	0.8210	0.057*	0.311 (6)
C36B	−0.0356 (13)	0.7063 (6)	0.9274 (8)	0.090*	0.311 (6)
H36D	−0.0864	0.6768	0.9173	0.135*	0.311 (6)
H36E	−0.0391	0.7232	0.9722	0.135*	0.311 (6)
H36F	0.0418	0.6949	0.9335	0.135*	0.311 (6)
C43B	0.3050 (6)	0.9326 (3)	0.4853 (4)	0.127 (2)*	0.528 (7)
H43C	0.3844	0.9228	0.4920	0.153*	0.528 (7)
H43D	0.2567	0.9093	0.4484	0.153*	0.528 (7)
C44B	0.2891 (8)	0.9820 (3)	0.4571 (5)	0.090*	0.528 (7)
H44D	0.3093	0.9833	0.4114	0.135*	0.528 (7)
H44E	0.3370	1.0057	0.4925	0.135*	0.528 (7)
H44F	0.2098	0.9918	0.4474	0.135*	0.528 (7)
C49B	0.7135 (4)	0.86634 (16)	0.6609 (2)	0.0706 (12)	0.550 (6)
H49C	0.7947	0.8651	0.6648	0.085*	0.550 (6)
H49D	0.6905	0.9027	0.6570	0.085*	0.550 (6)
C50B	0.6468 (8)	0.8387 (3)	0.5899 (4)	0.071 (2)*	0.550 (6)
H50C	0.5697	0.8320	0.5926	0.085*	0.550 (6)
H50D	0.6830	0.8052	0.5886	0.085*	0.550 (6)
C51B	0.6360 (7)	0.8667 (4)	0.5159 (5)	0.084 (3)*	0.550 (6)
H51C	0.5916	0.8455	0.4745	0.101*	0.550 (6)
H51D	0.5963	0.8996	0.5145	0.101*	0.550 (6)
C52B	0.7524 (7)	0.8760 (4)	0.5091 (5)	0.090*	0.550 (6)
H52D	0.7460	0.8937	0.4630	0.135*	0.550 (6)
H52E	0.7908	0.8432	0.5098	0.135*	0.550 (6)
H52F	0.7959	0.8970	0.5501	0.135*	0.550 (6)

### Atomic displacement parameters (Å^2^)

**Table:**

	*U*^11^	*U*^22^	*U*^33^	*U*^12^	*U*^13^	*U*^23^
Cl2	0.0748 (6)	0.0417 (4)	0.0787 (6)	0.0165 (4)	0.0400 (5)	0.0154 (4)
Cl3	0.0458 (5)	0.1302 (10)	0.1032 (9)	−0.0045 (6)	0.0382 (5)	−0.0416 (8)
Cl4	0.0657 (5)	0.0534 (5)	0.0494 (5)	0.0192 (4)	0.0145 (4)	0.0145 (4)
O1	0.0448 (11)	0.0461 (12)	0.0439 (12)	0.0083 (9)	0.0140 (9)	0.0151 (9)
O2	0.0321 (9)	0.0751 (15)	0.0379 (11)	0.0007 (10)	0.0183 (8)	0.0154 (10)
O3	0.0650 (16)	0.0857 (19)	0.0567 (16)	−0.0063 (14)	0.0347 (13)	−0.0134 (13)
O4	0.0454 (10)	0.0477 (11)	0.0271 (10)	−0.0056 (9)	0.0191 (8)	−0.0060 (8)
O5	0.0323 (9)	0.0290 (9)	0.0427 (11)	−0.0035 (8)	0.0129 (8)	−0.0016 (8)
O6	0.0375 (12)	0.0437 (13)	0.0898 (19)	0.0023 (10)	0.0099 (11)	0.0081 (12)
O7	0.0477 (11)	0.0374 (11)	0.0523 (13)	0.0020 (9)	0.0253 (10)	0.0121 (9)
O8	0.0304 (9)	0.0561 (12)	0.0397 (11)	−0.0098 (9)	0.0179 (8)	−0.0037 (9)
O9	0.0424 (12)	0.0867 (18)	0.0546 (14)	−0.0171 (12)	0.0182 (11)	−0.0215 (13)
O10	0.0383 (11)	0.0534 (13)	0.0516 (13)	−0.0091 (10)	0.0055 (9)	0.0080 (10)
O11	0.0391 (10)	0.0455 (11)	0.0353 (11)	0.0107 (9)	0.0115 (8)	0.0063 (9)
O12	0.0608 (14)	0.0762 (17)	0.0515 (14)	0.0352 (13)	0.0251 (11)	0.0118 (12)
C1	0.0355 (13)	0.0339 (14)	0.0308 (14)	−0.0045 (11)	0.0119 (11)	−0.0017 (11)
C2	0.0355 (13)	0.0352 (14)	0.0296 (14)	−0.0012 (11)	0.0068 (10)	0.0063 (11)
C3	0.0362 (13)	0.0446 (16)	0.0305 (14)	−0.0062 (12)	0.0120 (11)	0.0069 (12)
C4	0.0299 (12)	0.0458 (16)	0.0331 (14)	−0.0067 (12)	0.0139 (11)	0.0024 (12)
C5	0.0309 (12)	0.0321 (13)	0.0290 (13)	−0.0050 (10)	0.0105 (10)	−0.0006 (10)
C6	0.0328 (12)	0.0330 (14)	0.0297 (13)	−0.0032 (11)	0.0133 (10)	0.0015 (11)
C7	0.0295 (12)	0.0355 (14)	0.0271 (13)	−0.0035 (11)	0.0127 (10)	−0.0021 (10)
C8	0.0246 (11)	0.0329 (13)	0.0257 (12)	−0.0012 (10)	0.0094 (9)	−0.0014 (10)
C9	0.0261 (11)	0.0380 (14)	0.0259 (13)	−0.0002 (10)	0.0108 (9)	−0.0018 (10)
C10	0.0298 (12)	0.0356 (14)	0.0317 (14)	−0.0009 (11)	0.0107 (10)	−0.0091 (11)
C11	0.0254 (11)	0.0280 (13)	0.0349 (14)	−0.0020 (10)	0.0104 (10)	−0.0019 (10)
C12	0.0245 (11)	0.0348 (13)	0.0255 (12)	0.0018 (10)	0.0100 (9)	0.0010 (10)
C13	0.0272 (11)	0.0322 (13)	0.0271 (13)	−0.0013 (10)	0.0114 (10)	−0.0048 (10)
C14	0.0282 (12)	0.0328 (13)	0.0287 (13)	0.0006 (10)	0.0109 (10)	0.0037 (10)
C15	0.0294 (12)	0.0364 (14)	0.0223 (12)	−0.0026 (11)	0.0099 (9)	−0.0005 (10)
C16	0.0376 (13)	0.0365 (14)	0.0259 (13)	−0.0020 (11)	0.0124 (10)	0.0017 (11)
C17	0.0385 (14)	0.0411 (15)	0.0299 (14)	−0.0098 (12)	0.0146 (11)	0.0002 (11)
C18	0.0276 (12)	0.0501 (16)	0.0273 (13)	−0.0076 (12)	0.0130 (10)	−0.0027 (12)
C19	0.0283 (12)	0.0442 (15)	0.0254 (13)	−0.0014 (11)	0.0108 (10)	−0.0005 (11)
C20	0.0259 (11)	0.0374 (14)	0.0282 (13)	−0.0065 (11)	0.0106 (10)	0.0008 (11)
C21	0.0276 (12)	0.0442 (16)	0.0439 (16)	0.0013 (11)	0.0185 (11)	0.0017 (13)
C22	0.0291 (12)	0.0408 (15)	0.0395 (15)	0.0087 (11)	0.0153 (11)	0.0051 (12)
C23	0.0303 (13)	0.0391 (15)	0.0452 (17)	0.0020 (12)	0.0105 (12)	0.0061 (13)
C24	0.0329 (13)	0.0423 (16)	0.0414 (16)	0.0036 (12)	0.0043 (12)	0.0008 (13)
C25	0.0321 (13)	0.0390 (15)	0.0364 (15)	0.0111 (11)	0.0113 (11)	0.0055 (12)
C26	0.0289 (12)	0.0354 (14)	0.0396 (15)	0.0073 (11)	0.0141 (11)	0.0030 (12)
C27	0.0299 (12)	0.0388 (15)	0.0386 (15)	0.0037 (11)	0.0142 (11)	0.0027 (12)
C28	0.0380 (13)	0.0356 (14)	0.0373 (15)	0.0054 (12)	0.0156 (11)	0.0052 (12)
C29	0.060 (2)	0.063 (2)	0.048 (2)	0.0076 (17)	0.0150 (16)	0.0269 (17)
C30	0.0481 (17)	0.071 (2)	0.0404 (18)	0.0142 (17)	0.0257 (15)	0.0192 (16)
C32	0.0334 (13)	0.0366 (14)	0.0320 (14)	−0.0037 (11)	0.0093 (11)	−0.0028 (11)
C33	0.0373 (14)	0.0352 (15)	0.0464 (17)	−0.0051 (12)	0.0095 (12)	−0.0010 (12)
C37	0.0422 (15)	0.0614 (19)	0.0323 (15)	−0.0027 (14)	0.0178 (12)	−0.0134 (14)
C38	0.0394 (15)	0.0352 (15)	0.0447 (17)	0.0004 (12)	0.0155 (12)	−0.0013 (12)
C39	0.0533 (17)	0.0290 (15)	0.061 (2)	−0.0003 (13)	0.0213 (15)	0.0009 (13)
C40	0.0287 (12)	0.0514 (17)	0.0303 (14)	−0.0019 (12)	0.0099 (10)	−0.0004 (12)
C41	0.0470 (17)	0.093 (3)	0.0306 (16)	−0.0171 (18)	0.0107 (13)	0.0028 (16)
C42	0.080 (3)	0.128 (4)	0.0312 (19)	−0.032 (3)	0.0188 (18)	−0.018 (2)
C45	0.072 (2)	0.0436 (18)	0.056 (2)	0.0009 (16)	0.0337 (18)	0.0136 (15)
C46	0.0342 (14)	0.064 (2)	0.0407 (17)	−0.0113 (14)	0.0133 (12)	−0.0002 (15)
C47	0.0348 (16)	0.098 (3)	0.070 (2)	−0.0168 (18)	0.0225 (16)	−0.017 (2)
C48	0.0555 (18)	0.0491 (18)	0.0482 (18)	0.0004 (15)	0.0275 (15)	−0.0046 (14)
C53	0.064 (2)	0.059 (2)	0.069 (3)	−0.0237 (19)	−0.0126 (19)	0.0152 (19)
C54	0.0352 (14)	0.0433 (16)	0.0398 (16)	0.0067 (12)	0.0075 (12)	0.0014 (13)
C55	0.0470 (16)	0.0475 (17)	0.0371 (16)	0.0128 (14)	0.0084 (13)	0.0033 (13)
C56	0.0552 (17)	0.0404 (16)	0.0435 (17)	0.0006 (14)	0.0225 (14)	−0.0029 (13)
C57	0.087 (3)	0.0431 (19)	0.056 (2)	−0.0094 (18)	0.0287 (19)	−0.0064 (16)
C58	0.098 (3)	0.058 (2)	0.060 (2)	−0.009 (2)	0.025 (2)	−0.0034 (19)
C59	0.105 (3)	0.062 (3)	0.072 (3)	−0.023 (2)	0.009 (2)	0.001 (2)
C60	0.108 (4)	0.088 (4)	0.134 (5)	−0.027 (3)	0.003 (4)	0.031 (3)
Cl1A	0.0656 (16)	0.0933 (18)	0.0697 (19)	0.0057 (13)	0.0418 (13)	−0.0174 (15)
C31A	0.040 (4)	0.056 (5)	0.045 (4)	−0.001 (4)	0.026 (3)	−0.006 (4)
C34A	0.0429 (17)	0.051 (2)	0.093 (3)	−0.0074 (15)	0.0276 (18)	0.0000 (19)
C49A	0.097 (3)	0.073 (3)	0.054 (2)	−0.022 (2)	0.040 (2)	−0.0134 (19)
Cl1B	0.082 (2)	0.180 (4)	0.0565 (17)	0.013 (2)	0.0493 (15)	0.0147 (18)
C31B	0.041 (4)	0.114 (9)	0.041 (5)	0.006 (6)	0.020 (4)	0.010 (6)
C34B	0.0429 (17)	0.051 (2)	0.093 (3)	−0.0074 (15)	0.0276 (18)	0.0000 (19)
C49B	0.097 (3)	0.073 (3)	0.054 (2)	−0.022 (2)	0.040 (2)	−0.0134 (19)

### Geometric parameters (Å, °)

**Table:**

Cl2—C39	1.762 (3)	C40—H40A	0.9900
Cl3—C47	1.747 (4)	C40—H40B	0.9900
Cl4—C55	1.755 (3)	C41—C42	1.514 (5)
O1—C2	1.373 (3)	C41—H41A	0.9900
O1—C29	1.424 (4)	C41—H41B	0.9900
O2—C30	1.347 (4)	C42—C43A	1.484 (8)
O2—C4	1.413 (3)	C42—H42A	0.9900
O3—C30	1.181 (4)	C42—H42B	0.9900
O4—C9	1.370 (3)	C45—H45A	0.9800
O4—C37	1.429 (3)	C45—H45B	0.9800
O5—C38	1.349 (3)	C45—H45C	0.9800
O5—C11	1.415 (3)	C46—C47	1.514 (4)
O6—C38	1.189 (3)	C47—H47A	0.9900
O7—C16	1.366 (3)	C47—H47B	0.9900
O7—C45	1.427 (3)	C48—C49A	1.534 (5)
O8—C46	1.339 (4)	C48—H48A	0.9900
O8—C18	1.418 (3)	C48—H48B	0.9900
O9—C46	1.191 (4)	C53—H53A	0.9800
O10—C23	1.373 (3)	C53—H53B	0.9800
O10—C53	1.409 (4)	C53—H53C	0.9800
O11—C54	1.358 (3)	C54—C55	1.495 (4)
O11—C25	1.417 (3)	C55—H55A	0.9900
O12—C54	1.187 (3)	C55—H55B	0.9900
C1—C6	1.389 (4)	C56—C57	1.542 (5)
C1—C2	1.397 (4)	C56—H56A	0.9900
C1—C28	1.523 (4)	C56—H56B	0.9900
C2—C3	1.387 (4)	C57—C58	1.532 (5)
C3—C4	1.385 (4)	C57—H57A	0.9900
C3—H3	0.9500	C57—H57B	0.9900
C4—C5	1.385 (4)	C58—C59	1.543 (6)
C5—C6	1.400 (3)	C58—H58A	0.9900
C5—C7	1.516 (4)	C58—H58B	0.9900
C6—H6	0.9500	C59—C60	1.486 (7)
C7—C8	1.524 (3)	C59—H59A	0.9900
C7—C32	1.546 (4)	C59—H59B	0.9900
C7—H7	1.0000	C60—H60A	0.9800
C8—C13	1.395 (3)	C60—H60B	0.9800
C8—C9	1.401 (3)	C60—H60C	0.9800
C9—C10	1.387 (4)	Cl1A—C31A	1.758 (9)
C10—C11	1.382 (3)	C31A—H31A	0.9900
C10—H10	0.9500	C31A—H31B	0.9900
C11—C12	1.390 (3)	C34A—C35A	1.552 (7)
C12—C13	1.396 (3)	C34A—H34A	0.9900
C12—C14	1.522 (3)	C34A—H34B	0.9900
C13—H13	0.9500	C35A—C36A	1.561 (9)
C14—C15	1.527 (3)	C35A—H35A	0.9900
C14—C40	1.538 (4)	C35A—H35B	0.9900
C14—H14	1.0000	C36A—H36A	0.9800
C15—C20	1.380 (4)	C36A—H36B	0.9800
C15—C16	1.406 (4)	C36A—H36C	0.9800
C16—C17	1.395 (4)	C43A—C44A	1.497 (12)
C17—C18	1.375 (4)	C43A—H43A	0.9900
C17—H17	0.9500	C43A—H43B	0.9900
C18—C19	1.380 (4)	C44A—H44A	0.9800
C19—C20	1.406 (3)	C44A—H44B	0.9800
C19—C21	1.525 (4)	C44A—H44C	0.9800
C20—H20	0.9500	C49A—C50A	1.464 (9)
C21—C22	1.510 (4)	C49A—H49A	0.9900
C21—C48	1.527 (4)	C49A—H49B	0.9900
C21—H21	1.0000	C50A—C51A	1.564 (12)
C22—C27	1.400 (4)	C50A—H50A	0.9900
C22—C23	1.404 (4)	C50A—H50B	0.9900
C23—C24	1.387 (4)	C51A—C52A	1.576 (12)
C24—C25	1.383 (4)	C51A—H51A	0.9900
C24—H24	0.9500	C51A—H51B	0.9900
C25—C26	1.385 (4)	C52A—H52A	0.9800
C26—C27	1.404 (4)	C52A—H52B	0.9800
C26—C28	1.519 (4)	C52A—H52C	0.9800
C27—H27	0.9500	Cl1B—C31B	1.727 (10)
C28—C56	1.544 (4)	C31B—H31C	0.9900
C28—H28	1.0000	C31B—H31D	0.9900
C29—H29A	0.9800	C35B—C36B	1.456 (14)
C29—H29B	0.9800	C35B—H35C	0.9900
C29—H29C	0.9800	C35B—H35D	0.9900
C30—C31B	1.473 (11)	C36B—H36D	0.9800
C30—C31A	1.594 (10)	C36B—H36E	0.9800
C32—C33	1.525 (4)	C36B—H36F	0.9800
C32—H32A	0.9900	C44B—H44D	0.9800
C32—H32B	0.9900	C44B—H44E	0.9800
C33—C34A	1.536 (4)	C44B—H44F	0.9800
C33—H33A	0.9900	C50B—C51B	1.565 (10)
C33—H33B	0.9900	C50B—H50C	0.9900
C37—H37A	0.9800	C50B—H50D	0.9900
C37—H37B	0.9800	C51B—C52B	1.509 (11)
C37—H37C	0.9800	C51B—H51C	0.9900
C38—C39	1.512 (4)	C51B—H51D	0.9900
C39—H39A	0.9900	C52B—H52D	0.9800
C39—H39B	0.9900	C52B—H52E	0.9800
C40—C41	1.517 (4)	C52B—H52F	0.9800
			
C2—O1—C29	118.3 (2)	O7—C45—H45A	109.5
C30—O2—C4	119.1 (2)	O7—C45—H45B	109.5
C9—O4—C37	117.6 (2)	H45A—C45—H45B	109.5
C38—O5—C11	117.9 (2)	O7—C45—H45C	109.5
C16—O7—C45	117.3 (2)	H45A—C45—H45C	109.5
C46—O8—C18	119.3 (2)	H45B—C45—H45C	109.5
C23—O10—C53	117.4 (3)	O9—C46—O8	125.9 (3)
C54—O11—C25	116.7 (2)	O9—C46—C47	121.7 (3)
C6—C1—C2	118.0 (2)	O8—C46—C47	112.4 (3)
C6—C1—C28	120.6 (2)	C46—C47—Cl3	115.5 (2)
C2—C1—C28	121.2 (2)	C46—C47—H47A	108.4
O1—C2—C3	123.0 (2)	Cl3—C47—H47A	108.4
O1—C2—C1	116.6 (2)	C46—C47—H47B	108.4
C3—C2—C1	120.3 (2)	Cl3—C47—H47B	108.4
C4—C3—C2	119.0 (2)	H47A—C47—H47B	107.5
C4—C3—H3	120.5	C21—C48—C49A	112.2 (3)
C2—C3—H3	120.5	C21—C48—H48A	109.2
C3—C4—C5	123.6 (2)	C49A—C48—H48A	109.2
C3—C4—O2	118.0 (2)	C21—C48—H48B	109.2
C5—C4—O2	118.1 (2)	C49A—C48—H48B	109.2
C4—C5—C6	115.2 (2)	H48A—C48—H48B	107.9
C4—C5—C7	123.4 (2)	O10—C53—H53A	109.5
C6—C5—C7	121.2 (2)	O10—C53—H53B	109.5
C1—C6—C5	123.8 (2)	H53A—C53—H53B	109.5
C1—C6—H6	118.1	O10—C53—H53C	109.5
C5—C6—H6	118.1	H53A—C53—H53C	109.5
C5—C7—C8	111.7 (2)	H53B—C53—H53C	109.5
C5—C7—C32	110.8 (2)	O12—C54—O11	124.1 (3)
C8—C7—C32	113.3 (2)	O12—C54—C55	127.4 (3)
C5—C7—H7	106.9	O11—C54—C55	108.4 (2)
C8—C7—H7	106.9	C54—C55—Cl4	111.9 (2)
C32—C7—H7	106.9	C54—C55—H55A	109.2
C13—C8—C9	117.9 (2)	Cl4—C55—H55A	109.2
C13—C8—C7	123.5 (2)	C54—C55—H55B	109.2
C9—C8—C7	118.6 (2)	Cl4—C55—H55B	109.2
O4—C9—C10	123.7 (2)	H55A—C55—H55B	107.9
O4—C9—C8	115.7 (2)	C57—C56—C28	111.5 (2)
C10—C9—C8	120.6 (2)	C57—C56—H56A	109.3
C11—C10—C9	118.7 (2)	C28—C56—H56A	109.3
C11—C10—H10	120.6	C57—C56—H56B	109.3
C9—C10—H10	120.6	C28—C56—H56B	109.3
C10—C11—C12	123.8 (2)	H56A—C56—H56B	108.0
C10—C11—O5	117.0 (2)	C58—C57—C56	114.9 (3)
C12—C11—O5	119.1 (2)	C58—C57—H57A	108.5
C11—C12—C13	115.3 (2)	C56—C57—H57A	108.5
C11—C12—C14	120.5 (2)	C58—C57—H57B	108.5
C13—C12—C14	124.2 (2)	C56—C57—H57B	108.5
C8—C13—C12	123.6 (2)	H57A—C57—H57B	107.5
C8—C13—H13	118.2	C57—C58—C59	113.0 (3)
C12—C13—H13	118.2	C57—C58—H58A	109.0
C12—C14—C15	112.3 (2)	C59—C58—H58A	109.0
C12—C14—C40	113.7 (2)	C57—C58—H58B	109.0
C15—C14—C40	108.9 (2)	C59—C58—H58B	109.0
C12—C14—H14	107.2	H58A—C58—H58B	107.8
C15—C14—H14	107.2	C60—C59—C58	114.8 (4)
C40—C14—H14	107.2	C60—C59—H59A	108.6
C20—C15—C16	118.3 (2)	C58—C59—H59A	108.6
C20—C15—C14	120.4 (2)	C60—C59—H59B	108.6
C16—C15—C14	121.0 (2)	C58—C59—H59B	108.6
O7—C16—C17	123.6 (2)	H59A—C59—H59B	107.6
O7—C16—C15	116.3 (2)	C59—C60—H60A	109.5
C17—C16—C15	120.0 (2)	C59—C60—H60B	109.5
C18—C17—C16	119.0 (2)	H60A—C60—H60B	109.5
C18—C17—H17	120.5	C59—C60—H60C	109.5
C16—C17—H17	120.5	H60A—C60—H60C	109.5
C17—C18—C19	123.6 (2)	H60B—C60—H60C	109.5
C17—C18—O8	118.6 (2)	C30—C31A—Cl1A	107.5 (5)
C19—C18—O8	117.4 (2)	C30—C31A—H31A	110.2
C18—C19—C20	115.9 (2)	Cl1A—C31A—H31A	110.2
C18—C19—C21	123.6 (2)	C30—C31A—H31B	110.2
C20—C19—C21	120.0 (2)	Cl1A—C31A—H31B	110.2
C15—C20—C19	123.1 (2)	H31A—C31A—H31B	108.5
C15—C20—H20	118.4	C33—C34A—C35A	115.9 (3)
C19—C20—H20	118.4	C33—C34A—H34A	108.3
C22—C21—C19	110.7 (2)	C35A—C34A—H34A	108.3
C22—C21—C48	115.4 (2)	C33—C34A—H34B	108.3
C19—C21—C48	110.0 (2)	C35A—C34A—H34B	108.3
C22—C21—H21	106.8	H34A—C34A—H34B	107.4
C19—C21—H21	106.8	C34A—C35A—C36A	105.3 (5)
C48—C21—H21	106.8	C34A—C35A—H35A	110.7
C27—C22—C23	117.7 (3)	C36A—C35A—H35A	110.7
C27—C22—C21	123.8 (3)	C34A—C35A—H35B	110.7
C23—C22—C21	118.5 (2)	C36A—C35A—H35B	110.7
O10—C23—C24	123.2 (3)	H35A—C35A—H35B	108.8
O10—C23—C22	115.6 (2)	C35A—C36A—H36A	109.5
C24—C23—C22	121.1 (3)	C35A—C36A—H36B	109.5
C25—C24—C23	118.3 (3)	H36A—C36A—H36B	109.5
C25—C24—H24	120.9	C35A—C36A—H36C	109.5
C23—C24—H24	120.9	H36A—C36A—H36C	109.5
C24—C25—C26	124.2 (3)	H36B—C36A—H36C	109.5
C24—C25—O11	116.7 (3)	C42—C43A—C44A	112.2 (8)
C26—C25—O11	119.1 (2)	C42—C43A—H43A	109.2
C25—C26—C27	115.7 (2)	C44A—C43A—H43A	109.2
C25—C26—C28	119.9 (2)	C42—C43A—H43B	109.2
C27—C26—C28	124.4 (3)	C44A—C43A—H43B	109.2
C22—C27—C26	123.0 (3)	H43A—C43A—H43B	107.9
C22—C27—H27	118.5	C43A—C44A—H44A	109.5
C26—C27—H27	118.5	C43A—C44A—H44B	109.5
C26—C28—C1	111.2 (2)	H44A—C44A—H44B	109.5
C26—C28—C56	115.3 (2)	C43A—C44A—H44C	109.5
C1—C28—C56	110.2 (2)	H44A—C44A—H44C	109.5
C26—C28—H28	106.5	H44B—C44A—H44C	109.5
C1—C28—H28	106.5	C50A—C49A—C48	111.8 (5)
C56—C28—H28	106.5	C50A—C49A—H49A	109.3
O1—C29—H29A	109.5	C48—C49A—H49A	109.3
O1—C29—H29B	109.5	C50A—C49A—H49B	109.3
H29A—C29—H29B	109.5	C48—C49A—H49B	109.3
O1—C29—H29C	109.5	H49A—C49A—H49B	107.9
H29A—C29—H29C	109.5	C49A—C50A—C51A	111.8 (7)
H29B—C29—H29C	109.5	C49A—C50A—H50A	109.3
O3—C30—O2	125.7 (3)	C51A—C50A—H50A	109.3
O3—C30—C31B	116.3 (5)	C49A—C50A—H50B	109.3
O2—C30—C31B	116.9 (5)	C51A—C50A—H50B	109.3
O3—C30—C31A	133.8 (4)	H50A—C50A—H50B	107.9
O2—C30—C31A	99.7 (4)	C50A—C51A—C52A	117.7 (9)
C33—C32—C7	113.1 (2)	C50A—C51A—H51A	107.9
C33—C32—H32A	109.0	C52A—C51A—H51A	107.9
C7—C32—H32A	109.0	C50A—C51A—H51B	107.9
C33—C32—H32B	109.0	C52A—C51A—H51B	107.9
C7—C32—H32B	109.0	H51A—C51A—H51B	107.2
H32A—C32—H32B	107.8	C51A—C52A—H52A	109.5
C32—C33—C34A	113.7 (2)	C51A—C52A—H52B	109.5
C32—C33—H33A	108.8	H52A—C52A—H52B	109.5
C34A—C33—H33A	108.8	C51A—C52A—H52C	109.5
C32—C33—H33B	108.8	H52A—C52A—H52C	109.5
C34A—C33—H33B	108.8	H52B—C52A—H52C	109.5
H33A—C33—H33B	107.7	C30—C31B—Cl1B	116.4 (7)
O4—C37—H37A	109.5	C30—C31B—H31C	108.2
O4—C37—H37B	109.5	Cl1B—C31B—H31C	108.2
H37A—C37—H37B	109.5	C30—C31B—H31D	108.2
O4—C37—H37C	109.5	Cl1B—C31B—H31D	108.2
H37A—C37—H37C	109.5	H31C—C31B—H31D	107.3
H37B—C37—H37C	109.5	C36B—C35B—H35C	109.7
O6—C38—O5	124.8 (3)	C36B—C35B—H35D	109.7
O6—C38—C39	127.1 (3)	H35C—C35B—H35D	108.2
O5—C38—C39	108.1 (2)	C35B—C36B—H36D	109.5
C38—C39—Cl2	111.0 (2)	C35B—C36B—H36E	109.5
C38—C39—H39A	109.4	H36D—C36B—H36E	109.5
Cl2—C39—H39A	109.4	C35B—C36B—H36F	109.5
C38—C39—H39B	109.4	H36D—C36B—H36F	109.5
Cl2—C39—H39B	109.4	H36E—C36B—H36F	109.5
H39A—C39—H39B	108.0	H44D—C44B—H44E	109.5
C41—C40—C14	113.9 (2)	H44D—C44B—H44F	109.5
C41—C40—H40A	108.8	H44E—C44B—H44F	109.5
C14—C40—H40A	108.8	C51B—C50B—H50C	108.0
C41—C40—H40B	108.8	C51B—C50B—H50D	108.0
C14—C40—H40B	108.8	H50C—C50B—H50D	107.2
H40A—C40—H40B	107.7	C52B—C51B—C50B	109.2 (8)
C42—C41—C40	113.5 (3)	C52B—C51B—H51C	109.8
C42—C41—H41A	108.9	C50B—C51B—H51C	109.8
C40—C41—H41A	108.9	C52B—C51B—H51D	109.8
C42—C41—H41B	108.9	C50B—C51B—H51D	109.8
C40—C41—H41B	108.9	H51C—C51B—H51D	108.3
H41A—C41—H41B	107.7	C51B—C52B—H52D	109.5
C43A—C42—C41	118.0 (4)	C51B—C52B—H52E	109.5
C43A—C42—H42A	107.8	H52D—C52B—H52E	109.5
C41—C42—H42A	107.8	C51B—C52B—H52F	109.5
C43A—C42—H42B	107.8	H52D—C52B—H52F	109.5
C41—C42—H42B	107.8	H52E—C52B—H52F	109.5
H42A—C42—H42B	107.1		
			
C29—O1—C2—C3	10.5 (4)	C20—C19—C21—C22	−57.8 (3)
C29—O1—C2—C1	−168.7 (3)	C18—C19—C21—C48	−101.6 (3)
C6—C1—C2—O1	179.9 (2)	C20—C19—C21—C48	70.9 (3)
C28—C1—C2—O1	4.9 (4)	C19—C21—C22—C27	105.4 (3)
C6—C1—C2—C3	0.7 (4)	C48—C21—C22—C27	−20.3 (4)
C28—C1—C2—C3	−174.3 (3)	C19—C21—C22—C23	−75.4 (3)
O1—C2—C3—C4	−178.4 (3)	C48—C21—C22—C23	158.9 (2)
C1—C2—C3—C4	0.7 (4)	C53—O10—C23—C24	14.6 (4)
C2—C3—C4—C5	−0.8 (4)	C53—O10—C23—C22	−166.4 (3)
C2—C3—C4—O2	172.6 (3)	C27—C22—C23—O10	178.7 (2)
C30—O2—C4—C3	63.4 (4)	C21—C22—C23—O10	−0.5 (4)
C30—O2—C4—C5	−122.8 (3)	C27—C22—C23—C24	−2.3 (4)
C3—C4—C5—C6	−0.7 (4)	C21—C22—C23—C24	178.5 (2)
O2—C4—C5—C6	−174.1 (2)	O10—C23—C24—C25	−178.6 (2)
C3—C4—C5—C7	175.0 (2)	C22—C23—C24—C25	2.5 (4)
O2—C4—C5—C7	1.6 (4)	C23—C24—C25—C26	−0.9 (4)
C2—C1—C6—C5	−2.3 (4)	C23—C24—C25—O11	−178.8 (2)
C28—C1—C6—C5	172.8 (2)	C54—O11—C25—C24	−91.2 (3)
C4—C5—C6—C1	2.3 (4)	C54—O11—C25—C26	90.8 (3)
C7—C5—C6—C1	−173.5 (2)	C24—C25—C26—C27	−0.9 (4)
C4—C5—C7—C8	125.9 (3)	O11—C25—C26—C27	177.0 (2)
C6—C5—C7—C8	−58.6 (3)	C24—C25—C26—C28	178.0 (2)
C4—C5—C7—C32	−106.7 (3)	O11—C25—C26—C28	−4.1 (4)
C6—C5—C7—C32	68.7 (3)	C23—C22—C27—C26	0.4 (4)
C5—C7—C8—C13	102.2 (3)	C21—C22—C27—C26	179.7 (2)
C32—C7—C8—C13	−23.8 (3)	C25—C26—C27—C22	1.1 (4)
C5—C7—C8—C9	−79.0 (3)	C28—C26—C27—C22	−177.7 (2)
C32—C7—C8—C9	155.0 (2)	C25—C26—C28—C1	74.2 (3)
C37—O4—C9—C10	8.6 (4)	C27—C26—C28—C1	−107.0 (3)
C37—O4—C9—C8	−171.2 (2)	C25—C26—C28—C56	−159.5 (2)
C13—C8—C9—O4	178.7 (2)	C27—C26—C28—C56	19.3 (4)
C7—C8—C9—O4	−0.2 (3)	C6—C1—C28—C26	59.0 (3)
C13—C8—C9—C10	−1.0 (4)	C2—C1—C28—C26	−126.1 (3)
C7—C8—C9—C10	−179.9 (2)	C6—C1—C28—C56	−70.1 (3)
O4—C9—C10—C11	−179.4 (2)	C2—C1—C28—C56	104.8 (3)
C8—C9—C10—C11	0.3 (4)	C4—O2—C30—O3	6.3 (5)
C9—C10—C11—C12	1.3 (4)	C4—O2—C30—C31B	173.9 (6)
C9—C10—C11—O5	−175.4 (2)	C4—O2—C30—C31A	−164.8 (4)
C38—O5—C11—C10	−92.6 (3)	C5—C7—C32—C33	67.3 (3)
C38—O5—C11—C12	90.5 (3)	C8—C7—C32—C33	−166.2 (2)
C10—C11—C12—C13	−2.0 (4)	C7—C32—C33—C34A	69.2 (3)
O5—C11—C12—C13	174.6 (2)	C11—O5—C38—O6	−2.1 (4)
C10—C11—C12—C14	177.0 (2)	C11—O5—C38—C39	176.1 (2)
O5—C11—C12—C14	−6.4 (3)	O6—C38—C39—Cl2	−35.2 (4)
C9—C8—C13—C12	0.3 (4)	O5—C38—C39—Cl2	146.7 (2)
C7—C8—C13—C12	179.1 (2)	C12—C14—C40—C41	175.2 (2)
C11—C12—C13—C8	1.2 (3)	C15—C14—C40—C41	−58.8 (3)
C14—C12—C13—C8	−177.8 (2)	C14—C40—C41—C42	177.8 (3)
C11—C12—C14—C15	75.8 (3)	C40—C41—C42—C43A	−170.1 (4)
C13—C12—C14—C15	−105.3 (3)	C18—O8—C46—O9	−3.6 (5)
C11—C12—C14—C40	−160.0 (2)	C18—O8—C46—C47	175.7 (3)
C13—C12—C14—C40	18.9 (3)	O9—C46—C47—Cl3	−174.7 (3)
C12—C14—C15—C20	58.4 (3)	O8—C46—C47—Cl3	5.9 (4)
C40—C14—C15—C20	−68.4 (3)	C22—C21—C48—C49A	−172.4 (3)
C12—C14—C15—C16	−128.4 (2)	C19—C21—C48—C49A	61.5 (3)
C40—C14—C15—C16	104.8 (3)	C25—O11—C54—O12	−3.1 (4)
C45—O7—C16—C17	12.5 (4)	C25—O11—C54—C55	175.5 (2)
C45—O7—C16—C15	−166.6 (3)	O12—C54—C55—Cl4	−16.3 (4)
C20—C15—C16—O7	179.1 (2)	O11—C54—C55—Cl4	165.2 (2)
C14—C15—C16—O7	5.8 (4)	C26—C28—C56—C57	175.6 (3)
C20—C15—C16—C17	−0.1 (4)	C1—C28—C56—C57	−57.5 (3)
C14—C15—C16—C17	−173.4 (2)	C28—C56—C57—C58	−174.5 (3)
O7—C16—C17—C18	−177.7 (2)	C56—C57—C58—C59	−173.0 (4)
C15—C16—C17—C18	1.4 (4)	C57—C58—C59—C60	−62.1 (6)
C16—C17—C18—C19	−0.7 (4)	O3—C30—C31A—Cl1A	25.1 (9)
C16—C17—C18—O8	172.3 (2)	O2—C30—C31A—Cl1A	−164.9 (5)
C46—O8—C18—C17	73.0 (3)	C31B—C30—C31A—Cl1A	−31.5 (12)
C46—O8—C18—C19	−113.6 (3)	C32—C33—C34A—C35A	−174.4 (4)
C17—C18—C19—C20	−1.3 (4)	C33—C34A—C35A—C36A	−76.9 (5)
O8—C18—C19—C20	−174.3 (2)	C41—C42—C43A—C44A	160.2 (7)
C17—C18—C19—C21	171.5 (3)	C21—C48—C49A—C50A	169.0 (6)
O8—C18—C19—C21	−1.5 (4)	C48—C49A—C50A—C51A	−176.2 (7)
C16—C15—C20—C19	−2.0 (4)	C49A—C50A—C51A—C52A	−42.7 (13)
C14—C15—C20—C19	171.3 (2)	O3—C30—C31B—Cl1B	−39.3 (10)
C18—C19—C20—C15	2.7 (4)	O2—C30—C31B—Cl1B	151.9 (6)
C21—C19—C20—C15	−170.4 (2)	C31A—C30—C31B—Cl1B	98.5 (19)
C18—C19—C21—C22	129.7 (3)		
